# Medium optimization for high mycelial soluble protein content of *Ophiocordyceps sinensis* using response surface methodology

**DOI:** 10.3389/fmicb.2022.1055055

**Published:** 2022-12-09

**Authors:** Chu-Yu Tang, Jie Wang, Xin Liu, Jian-Bo Chen, Jing Liang, Tao Wang, Wayne Roydon Simpson, Yu-Ling Li, Xiu-Zhang Li

**Affiliations:** ^1^State Key Laboratory of Plateau Ecology and Agriculture, Qinghai Academy of Animal and Veterinary Sciences, Qinghai University, Xining, China; ^2^AgResearch Grasslands Research Centre, Palmerston North, New Zealand

**Keywords:** *Ophiocordyceps sinensis*, mycelium, response surface methodology, transcriptome, soluble protein content

## Abstract

*Ophiocordyceps sinensis* is widely utilized due to its pharmaceutical value. Mycelial protein forms a key active component of *O. sinensis* and determines the medicinal potential of fungus. Here, we describe the development of an optimized fermentation medium to obtain more mycelial soluble protein from *O. sinensis* using response surface methodology (RSM) and investigate the increased mycelial protein content using transcriptomics. The maximum mycelial protein content of 2.11% was obtained using a medium consisting of 20% beef broth, 0.10% peptone, 2% glucose, 0.15% yeast extract, 0.20% KH_2_PO_4_, and 0.02% MgSO_4_. Transcriptome analysis identified 790 differentially expressed genes (DEGs), including 592 up-regulated genes and 198 down-regulated genes, optimisation resulted in more up-regulated genes. The main DEGs were enriched in metabolic pathways, ABC transporters, starch and sucrose metabolism, tyrosine metabolism, and glutathione metabolism. In addition, some DEGs associated with mycelial protein enhancement such as tyrosinase (*TYR*), glutathione S-transferase (*GST*), glutamine synthetase (*glnA*), and β-glucosidase may contribute to increased mycelial protein content. Real-time quantitative PCR (RT-qPCR) was used to confirm gene expression and the results support the accuracy of RNA-Seq and DEG analysis. This study provides an optimized fermentation method for enhancing the mycelial protein content of *O. sinensis* and a reference for the effective development of *O. sinensis* protein.

## Introduction

Chinese cordyceps or ‘Dong Chong Xia Cao’ is rare in nature. It is a complex of the larvae of endemic ghost moths (Hepialidae) parasitized by *Ophiocordyceps sinensis* and distributed on hillsides of the Tibetan Plateau at altitudes of 3,000–5,000 meters (Qiu et al., 2020; Li et al., 2021). The growth niche presents a hostile environment that leads to unstable Chinese cordyceps production and a variable metabolism that affects the production of active metabolites. This contingent production of Chinese cordyceps contributes to the fungus being extremely expensive and overexploited (Wei et al., 2021). The scarcity and high commercial value of Chinese cordyceps have stimulated the demand for Cordyceps-like products (He, 2018). Contemporary pharmacological studies indicated that the mycelial products of *Ophiocordyceps sinensis*, produced using fermentation technologies, have similar pharmaceutical values to Chinese cordyceps demonstrating influence over the respiratory, cardiovascular, immune systems and the treatment of chronic diseases (Dong et al., 2009; Zhang et al., 2012, 2020; Li et al., 2021; Yuan et al., 2022; Wu et al., 2022). This method of production has gradually been accepted as an alternative to the harvesting of naturally occurring fungus (Hsieh et al., 2005; Li et al., 2011; Wang et al., 2014). In terms of pharmacological activity, studies have shown that the substances with immune and antioxidant effects are Cordyceps proteins (Kong et al., 2021). With progressive research on Cordyceps proteins, the basic structures of the peptides have been elucidated (Jia et al., 2005) and most soluble proteins have been shown to be mostly functional proteins in *O. sinensis*, whose diversity is generally associated with the versatility of the medicinal fungal effects (Ren et al., 2013). Many of the proteins are extracellular or intracellular proteases, additionally a protein has been identified that in the form of a novel protein-binding polysaccharide HS002-II demonstrates significant immunomodulatory activity (He et al., 2013; Rakhee et al., 2021). A previous study has identified at least two fibrinolytic enzymes from *O. sinensis* mycelium with research implications for the development of drugs for thrombotic diseases (Zang et al., 2020). With the exception of adenosine, signature proteins differ from other Cordyceps genera and can used as markers in identification and quality determination assays (Chan et al., 2018; Qian et al., 2019). The elevation of *O. sinensis* mycelial protein content and examination of the factors influencing efficacy are basic requirements for the exploitation of Cordyceps mycoproteins.

The Plackett-Bruman design (PBD) is a preferred way to screen for significant biological factors and is often used in conjunction with the response surface methodology (RSM) containing the Central Composite Design (CCD) and the Box–Behnken design (BBD), where the BBD includes all global designs and only requires three layers of factors to be operational (Addelman, 1979; Dong et al., 2009; Zhang et al., 2022). In fermentation process research, RSM technology has been used to optimize all influencing parameters collectively and improve product content (Guo et al., 2018). It can be used to evaluate the relative importance of several influencing factors. At the same time, the variability in the fermentation process can be reduced, the output response can be monitored, and the response of the output to the nominal and target requirements can be closely confirmed, which has the advantages of reducing development time and overall cost (Elibol, 2002). Most of the designs have been successfully applied to optimize the extraction method and medium. Hsieh et al. optimized the medium for obtaining high-content *Cordyceps sinensis* polysaccharide and found that the optimal polysaccharide production medium was composed of 6.17% sucrose, 0.53% corn steep powder, 0.5% (NH_4_)_2_ HPO_4_, and 0.15% KH_2_PO_4_ at pH 4.44 (Hsieh et al., 2005). However, there is no report concerning the optimization of mycelial protein in media for *O. sinensis*.

The transcriptome is the basis for the investigation of gene function and structure. It has become increasingly popular in medicine, animals, plants, and fungi since it can rapidly reveal the molecular mechanisms of physiological changes in organisms and can inform subsequent proteomic and metabolomic studies (Hao et al., 2017; Wan et al., 2022; Wei et al., 2022). Previous studies have utilized transcriptomic analysis of *O. sinensis* to determine genes involved in key pathways of fruiting body development (Xiang et al., 2014; Tong et al., 2020). It has been demonstrated that genes such as nucleoside diphosphate kinase gene (*ndk*) and β subunit of the fatty acid synthase gene (*cel-2*) play a crucial role in the development of both growth and fruiting body development (Tong et al., 2020). Studies examining DEGs have also shown that some key genes related to fatty acid metabolism are upregulated in Chinese cordyceps with unsaturated fatty acid production being high in Chinese Cordyceps compared to cultivated fungus by transcriptome analysis (Zhang et al., 2021). Current information regarding the transcriptome of *O. sinensis* following optimization of the culture medium is scant. The results of this study provide a valuable reference for the cultivation and utilization of high mycelial protein of *O. sinensis*.

## Materials and methods

### Fungal isolation and shake flask fermentation

The mycelia of *O. sinensis* (TOS-1) was originally isolated from Chinese cordyceps collected from Zaduo County (E 95°8′23″, N 32°47′38″, H: 4601 m), Qinghai Province, China and deposited at the State Key Laboratory of Plateau Ecology and Agriculture, Qinghai Academy of Animal and Veterinary Sciences, Qinghai University. The sequence of TOS-1 was submitted to the National Center for Biotechnology Information with the corresponding serial number: OP379526. Phylogenetic analysis confirmed that the characteristic strain of this species was *O. sinensis*. The strain was activated and inoculated in a new 300 ml conical flask containing 150 ml of fermentation medium at an inoculation rate of 10% (v/v). It was cultured on a rotary shaker at 18°C for 70 d at 135 rpm using a fermentation medium containing (1:2) 50% beef broth, 0.50% peptone, 0.15% yeast powder, 3% glucose, 0.02% MgSO_4_ and 0.20% KH₂PO_4_. At the conclusion of liquid culture, the mycelium balls were harvested by centrifugation for 20 min at 6,000 ×*g*, washed with deionized water 3–4 times, the clean mycelium was placed in an incubator at 24 ~ 26°C to keep it dry and its weight constant and stored at −80°C for further analysis.

### Extraction of mycelial protein

The mycelial protein of *O. sinensis* was extracted using Minute™ Total Protein Extraction Kit for Microbes with Thick Cell Walls (Invent Biotechnologies Inc., United States) and store at −20°C for further analysis. Bradford’s assay was performed to determine the mycelial soluble protein content of the *O. sinensis* (Bradford, 1976). Bovine serum albumin (BSA) was used as the standard. The protein concentration was calculated from the absorbance and the final protein content was determined according to the following equation:


$$\mathrm{Protein}\;\mathrm{content}\;\left(\%\right)=\frac{C*V}{M}\;\times 100\%$$

where C (mg/mL) is the mycelial protein concentration, V (mL) is the volume of the extract, and M (mg) is the mass of the mycelium.

### Plackett-Burman design

Screening and evaluation of important media components affecting mycelial protein content of *O. sinensis* was performed using a Plackett-Burman design. In this study, all treatments based on the principal components of six variables (beef broth, peptone, yeast extract, glucose, KH₂PO_4_, MgSO_4_) of the medium were performed in triplicate according to the designed matrix, 10% (v/v) strain suspension was inoculated in each 150 ml Erlenmeyer flask containing sterilized substrate, flasks were incubated at 18°C for 24 days. This designed matrix using the equation includes two levels, low (−1) and high (+1), shown in (Supplementary Table S1).


$$Y\;\left(\%\right)=\beta_{0}+\sum \mathrm{\beta iXi}\left(i=1,\ldots k\right)$$

where *Y* represents the response, *β*_0_ represents the constant, and *β_i_* is the regression coefficient, *X* is the independent variable and k is the number of variables.

### Path of steepest ascent method

The three variables affecting mycelial protein content were selected based on the results of the PBD design, and the optimal levels of the variables were selected using the path of the steepest ascent method. The optimal region with the highest content was approached based on the positive and negative effects of the key variables and reasonable step size was designed. The central point of RMS can be obtained by the path of the steepest ascent method. The direction change and the step size range were designed as shown in Supplementary Table S2.

### Box–Behnken design

Three important factors, beef broth, peptone, and glucose, were determined by PBD to affect the mycelial protein of *O. sinensis*. Each variable was studied independently at three levels (−1, 0, 1). 17 treatments with details of variables and levels are shown in Supplementary Table S3. Independent variables were calculated using quadratic polynomial equations:


$$\begin{array}{l} Y\;\left(\%\right)=\beta_{0}+\beta_{1}X_{1}+\beta_{2}X_{2}+\beta_{3}X_{3}+\beta_{11}X_{1}^{2}+\beta_{22}X_{2}^{2}\\ \;+\beta_{33}X_{3}^{2}+\beta_{12}X_{1}X_{2}+\beta_{13}X_{1}X_{3}+\beta_{23}X_{2}X_{3} \end{array}$$

where *Y* represents the response, *β*_0_ represents the constant, and *β_i_*, *β_ii_*, and *β_ij_* are the regression coefficients of the three main effects, respectively.

### Statistical analysis

All data were analyzed by Design-Expert 11.0 and GraphPad Prism 8.0.2 was used to plot the data. All experiments are expressed as mean ± standard error (SEM).

### Sample collection

The mycelia of *O. sinensis* optimized medium (BBD) and unoptimized medium (CK) samples were obtained under the culture conditions of 24 days at 18°C. Then, the clean mycelium was placed in an incubator at 24 ~ 26°C to keep it dry and its weight constant. Three replicate samples were prepared for each treatment. After collection, all samples were immediately frozen in liquid nitrogen at −80°C prior to RNA isolation.

### RNA isolation and sequencing

Total RNA was isolated using Trizol (Invitrogen, United States) according to the manufacturer’s instructions. RNA degradation and contamination was monitored on 1% agarose gels, and the concentration and integrity of RNA were measured using Qubit^®^ RNA Assay Kit in Qubit^®^ 2.0 Flurometer (Life Technologies, CA, United States) and the RNA Nano 6000 Assay Kit of the Bioanalyzer 2100 system (Agilent Technologies, CA, United States). Sequencing libraries were generated using NEBNext^®^ Ultra^TM^ RNA Library Prep Kit for Illumina^®^ (NEB, United States) following the manufacturer’s recommendations. The quality of the library was assessed using the Agilent Bioanalyzer 2100 system. The cDNA libraries were sequenced on an Illumina NovaSeq 6000 platform (Metware Biological Science and Technology Co., Ltd., Wuhan, China) and 150 bp paired-end reads were generated. The original data were processed to remove adapter sequences, ploy-N sequences, low-quality reads, and very short-length reads. All subsequent analyses are based on clean reads.

### Data analysis and functional annotation of differentially expressed genes

The reference genome information of *O. sinensis* was downloaded from^1^ (Shu et al., 2020). Clean reads of each sample with three replicates were aligned to the reference genome using HISAT2 (v2.1.0). Gene alignment was calculated using feature Counts (version v1.6.2) along with Fragments Per Kilobase of exon model per Million mapped fragments (FPKM) of each gene based on the gene length. DESeq2 (version v1.22.1) was used to analyze the differential expression between the two groups, and the *p* value was corrected using the Benjamini & Hochberg method. Differentially expressed genes (DEGs) were identified using |log_2_FC (log_2_fold-change)| ≥1 and false discovered rate (FDR) <0.05 as statistically significant. Enrichment tests were performed based on Gene Ontology (GO) functions and Kyoto Encyclopedia of Genes and Genomes (KEGG^2^) pathways. The raw Illumina sequencing results of *O. sinensis* were deposited in the NCBI Sequence Read Archive (SRA) with the accession number: (PRJNA874172).

### Expression analysis by real-time quantitative PCR

Thirteen DEGs were arbitrarily selected for transcriptome sequencing analysis and validated by RT-qPCR experiments in triplicate. The *ACT1* gene was used to normalize the expression level. The specific primer sequences are listed in Supplementary Table S4. The reaction system contains 10 μl of Hieff^®^ RT-qPCR SYBR Green Master Mix (Low Rox Plus) (Yeasen Biotechnology Co., Shanghai, China), 0.4 μl of each primer (0.2 μM), 1 μl of cDNA, and 8.2 μl of RNase-free H_2_O. The cycling conditions were as follows: 95°C for 5 min, 40 cycles of 95°C for 10 s and 60°C for 30 s. Analysis of relative gene expression levels by the 2^−ΔΔCt^ method. The GraphPad Prism 8.0.2 (version 8.0.2, GraphPad Software, Inc.) was used for statistical analysis.

## Results

### Plackett-Burman design

PBD was used to screen for medium variables affecting the mycelium, 6 variables were investigated for their effect. The 12 treatments and their correspondences are shown in Table 1. Design Expert 11.0 was used to fit the treatment data in Table 1, the factor contribution rate and variance analysis results are shown in Table 2. *p* < 0.05 suggests significant model terms. The corrected coefficient of determination *R*^2^_adj_ was 0.8853, indicating that 88.53% of the variability of the treatment data could be explained by the model. The effect of beef broth (X_1_), peptone (X_2_), and glucose (X_4_) on mycelial protein content was highly significant (*p* < 0.01). Therefore, X_1_, X_2_, and X_4_ can be considered the main factors affecting mycelia protein content. The following model equation has been obtained for mycelia protein content (*Y*). Among the significant factors, the regression coefficients of factors X_1_ and X_4_ are negative, indicating that their effect on mycelial protein content is negative, and X_2_ is positive, suggesting that the addition of X_1_ and X_4_ may be appropriately reduced and the content of X_2_ may be increased in subsequent studies. According to the size analysis of the contribution rate and correlation coefficient in Table 2, the secondary factors X_3_, X_5_ and X_6_ were no longer added in the subsequent treatments, and it was determined that the addition amount of the original medium was 0.15, 0.2, 0.02%, respectively.


$$\begin{array}{l} Y\;\left(\%\right)=1.64-0.0608X_{1}+0.0925X_{2}-0.0142X_{3}\\ \;-0.0808X_{4}-0.0025X_{5}+0.0158X_{6} \end{array}$$

**Table 1:** Plackett-Burman experimental design matrix for screening composition of the growth medium.

Treatment	Level and concentration of variable (%)						Protein content (%)		
	X_1_	X_2_	X_3_	X_4_	X_5_	X_6_			
	Beef broth	Peptone	Yeast extract	Glucose	KH₂PO_4_	MgSO_4_	Actual value	Predicted value	Residual
1	1 (50)	1 (0.70)	1 (0.25)	−1 (1)	−1 (0.10)	−1 (0.01)	1.75 ± 0.01	1.73	0.02
2	1 (50)	−1 (0.30)	1 (0.25)	1 (4)	−1 (0.10)	1 (0.04)	1.43 ± 0.04	1.41	0.02
3	1 (50)	1 (0.70)	−1 (0.10)	1 (4)	1 (0.40)	1 (0.04)	1.61 ± 0.01	1.62	−0.01
4	−1 (30)	−1 (0.30)	1 (0.25)	−1 (1)	1 (0.40)	1 (0.04)	1.64 ± 0.03	1.69	−0.05
5	1 (50)	−1 (0.30)	−1 (0.10)	−1 (1)	1 (0.40)	−1 (0.01)	1.54 ± 0.01	1.57	−0.03
6	1 (50)	−1 (0.30)	1 (0.25)	1 (4)	1 (0.40)	−1 (0.01)	1.40 ± 0.04	1.38	0.02
7	−1 (30)	1 (0.70)	1 (0.25)	−1 (1)	1 (0.40)	1 (0.04)	1.93 ± 0.06	1.88	0.05
8	−1 (30)	−1 (0.30)	−1 (0.10)	1 (4)	−1 (0.10)	1 (0.04)	1.58 ± 0.06	1.56	0.02
9	−1 (30)	1 (0.70)	1 (0.25)	1 (4)	−1 (0.10)	−1 (0.01)	1.63 ± 0.04	1.69	−0.06
10	1 (50)	1 (0.70)	−1 (0.10)	−1 (1)	−1 (0.10)	1 (0.04)	1.77 ± 0.01	1.79	−0.02
11	−1 (30)	−1 (0.30)	−1 (0.10)	−1 (1)	−1 (0.10)	−1 (0.01)	1.72 ± 0.06	1.69	0.03
12	−1 (30)	1 (0.70)	−1 (0.10)	1 (4)	1 (0.40)	−1 (0.01)	1.73 ± 0.08	1.71	0.02

**Table 2:** Analysis of the significance of PB design.

Source	Sum of squares	*df*	Mean square	*F*-value	*p*-value
X_1_ (beef broth)	0.0444	1	0.0444	17.47	0.0087**
X_2_ (peptone)	0.1027	1	0.1027	40.4	0.0014**
X_3_ (yeast extract)	0.0024	1	0.0024	0.9475	0.3751
X_4_ (glucose)	0.0784	1	0.0784	30.85	0.0026**
X_5_ (KH₂PO_4_)	0.0001	1	0.0001	0.0295	0.8703
X_6_ (MgSO_4_)	0.003	1	0.003	1.18	0.3263

ANOVA using design-expert 11.0. **significant at *p* < 0.01.

### Path of steepest ascent method

According to Table 3, it was found that the highest mycelial protein content was reached in the second group of treatments, so the corresponding beef broth content (30%), peptone (0.3%), and glucose (2%) in this group were used as the central points for the subsequent response surface experiments.

**Table 3:** Design and results of steepest ascent method.

Treatment	Beef broth (%)	Peptone (%)	Yeast extract (%)	Glucose (%)	KH₂PO_4_ (%)	MgSO_4_ (%)	Protein content (%)
1	20	0.1	0.15	1	0.2	0.02	2.09 ± 0.03
2	30	0.3	0.15	2	0.2	0.02	2.17 ± 0.06
3	40	0.5	0.15	3	0.2	0.02	2.06 ± 0.06
4	50	0.7	0.15	4	0.2	0.02	2.02 ± 0.04
5	60	0.9	0.15	5	0.2	0.02	1.75 ± 0.11

### BBD model fitting and response surface analysis

Multiple regressions were fitted to the data in Table 4 using Design Expert 11.0 software to construct regression models for the extraction process parameters. With *O. sinensis* mycelial protein content as the response variable, beef broth, peptone, and glucose as the test variables, the response and test variables can be developed into a quadratic multinomial regression prediction model equation.


$$\begin{array}{l} Y\;\left(\%\right)=2.07+0.0275X_{1}-0.04X_{2}\\ \;+0.18X_{3}-0.045X_{1}^{2}+0.04X_{2}^{2}-0.26X_{3}^{2}\\ \;+0.03X_{1}X_{2}-0.05X_{1}X_{3}+0.095X_{2}X_{3} \end{array}$$

**Table 4:** Box–Behnken design with experimental and predicted values of mycelial protein content.

Run	Coded levels of variables			Protein content (%)		
	X_1_	X_2_	X_3_	Actual value	Predicted value	Residual
1	0	1	−1	1.54 ± 0.12	1.54	0.00
2	0	0	0	2.03 ± 0.02	2.07	−0.04
3	−1	0	−1	1.48 ± 0.01	1.51	−0.03
4	0	−1	−1	1.84 ± 0.06	1.81	0.04
5	−1	0	1	1.98 ± 0.08	1.97	0.01
6	0	0	0	2.08 ± 0.03	2.07	0.01
7	−1	1	0	1.99 ± 0.13	1.97	0.02
8	1	0	−1	1.65 ± 0.01	1.66	−0.01
9	1	0	1	1.95 ± 0.02	1.92	0.03
10	0	0	0	2.09 ± 0.03	2.07	0.02
11	1	1	0	2.09 ± 0.09	2.08	0.01
12	0	1	1	2.05 ± 0.01	2.09	−0.04
13	0	−1	1	1.97 ± 0.03	1.98	−0.01
14	0	0	0	2.07 ± 0.04	2.07	0.00
15	0	0	0	2.08 ± 0.01	2.07	0.01
16	1	−1	0	2.08 ± 0.11	2.1	−0.02
17	−1	−1	0	2.10 ± 0.08	2.11	−0.01

The results are shown in Table 5 that the model *p* < 0.0001 and lacks a fit term *p* = 0.1393, which indicates a significant regression and an insignificant misfit model. The correlation coefficient of the model *R*^2^ = 0.988 indicates a good fit for the model. The correction coefficient of determination *R*^2^_adj_ = 0.9725 indicates that approximately 97.25% of the variation in response values (*Y*) can be explained by the model. The model coefficient of variation (CV) value of 1.7% < 10% indicates that the Box–Behnken test has good confidence and accuracy and the model is appropriate. The linear coefficients (X_2_ and X_3_), cross-product coefficients (X_1_X_3_ and X_2_X_3_), and quadratic term coefficients (X_1_^2^, X_2_^2^, and X_3_^2^) were significant (*p* < 0.05), whereas the other terms coefficients were not significant (*p* > 0.05). The *F* values confirmed the order of factors affecting mycelial protein content as X_3_ > X_2_ > X_1_ and the order effect of interactions as X_2_X_3_ > X_1_X_3_ > X_1_X_2_. Design Expert 11.0 software was applied to optimize the parameters of the proposed model, and the maximum mycelial protein content was 2.11% when the concentration of beef broth was 20%, peptone was 0.10%, and glucose was 2%. The optimized medium formulation was finally determined as 20% beef broth, 0.10% peptone, 2% glucose, 0.15% yeast extract, 0.20% KH₂PO_4_, 0.02% MgSO_4_.

**Table 5:** ANOVA of the fitted polynomial quadratic model.

Source	Sum of squares	*df*	Mean square	*F*-value	*p*-value
Model	0.6292	9	0.0699	63.97	< 0.0001**
X1-beef broth	0.006	1	0.006	5.54	0.0509
X2-peptone	0.0128	1	0.0128	11.71	0.0111*
X3-glucose	0.2592	1	0.2592	237.18	< 0.0001**
X_1_X_2_	0.0036	1	0.0036	3.29	0.1124
X_1_X_3_	0.01	1	0.01	9.15	0.0193*
X_2_X_3_	0.0361	1	0.0361	33.03	0.0007**
X_1_^2^	0.0085	1	0.0085	7.8	0.0268*
X_2_^2^	0.0067	1	0.0067	6.16	0.042*
X_3_^2^	0.2846	1	0.2846	260.45	< 0.0001**
Residual	0.0077	7	0.0011		
Lack of fit	0.0055	3	0.0018	3.3	0.1393
Pure error	0.0022	4	0.0006		
Cor total	0.6368	16			
*R* ^2^	0.988		Adj *R*^2^	0.9725	C.V.% = 1.7

ANOVA using design-expert 11.0. *Significant at 0.01 < *p* < 0.05, **significant at *p* < 0.01.

**Figure 1:**
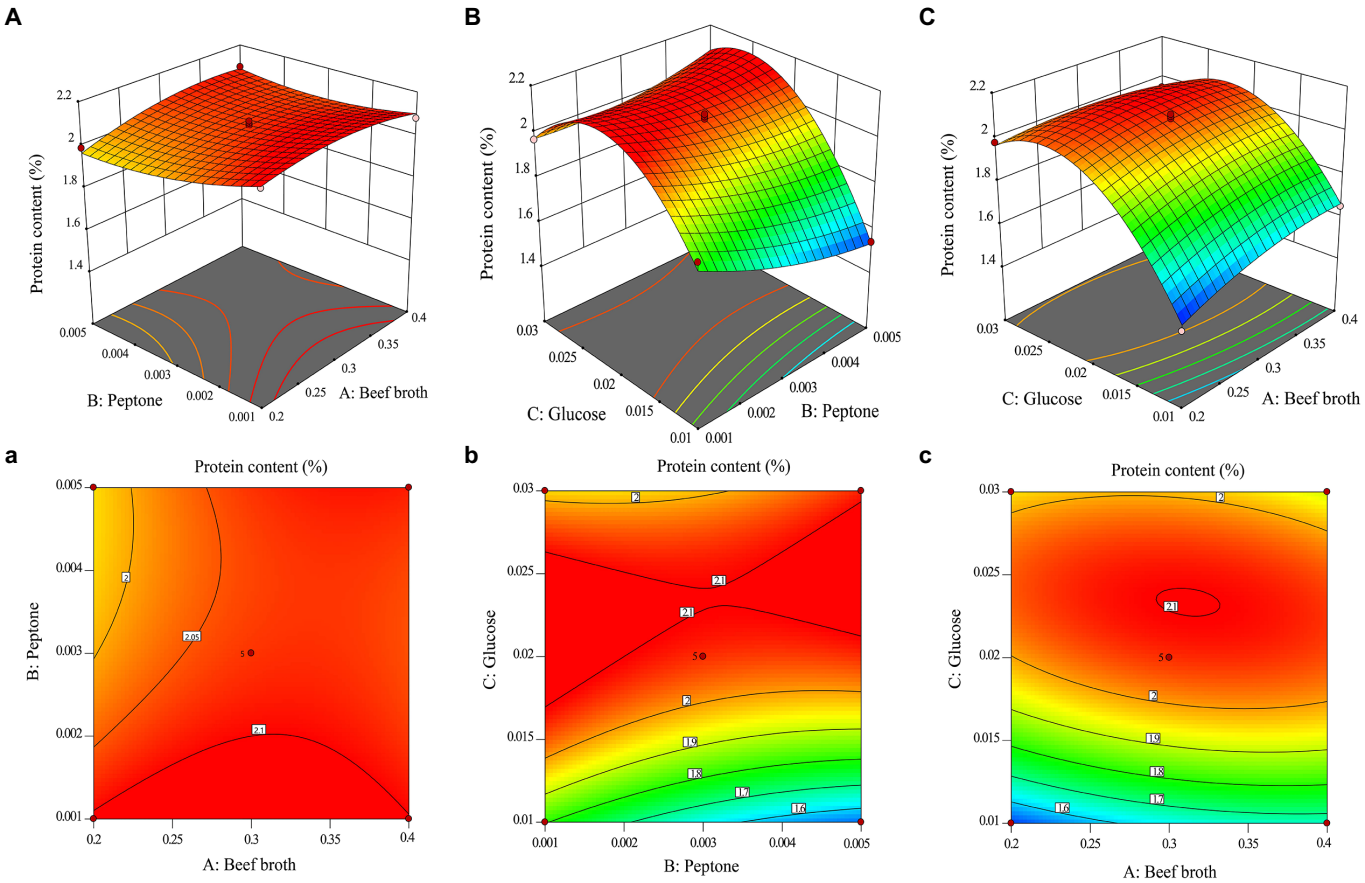
3D response surface and corresponding contour line of mycelial protein content: **(A)** Effect of beef broth and peptone addition on mycelial protein content; **(B)** Effect of peptone and glucose addition on mycelial protein content; **(C)** Effect of beef broth and glucose addition on mycelial protein content.

To visually analyze the magnitude of the effect of the other two factors and their interactions on the response values, one beef broth (X_1_), peptone (X_2_), and glucose (X_3_) was fixed at the centroid level (0 levels), respectively. The contours of the regression model and their response surfaces are shown in Figure 1. It can be seen from the figure that the slope of B and C is steeper than that of A, indicating that the significant interaction between B (X_2_ and X_3_) and C (X_1_ and X_3_). The average value of three replicate experiments was obtained and the mycelial protein content of *O. sinensis* mycelia was (2.03 ± 0.01) %, which was extremely close to the predicted value of 2.11% and fitted well with the predicted value, indicating that the above model can better predict the mycelial protein content of mycelia production.

### Transcriptome sequencing results and sequence assembly

To investigate the expression changes before (CK) and after (BBD) optimization of *O. sinensis*, this study performed a transcriptomic study with three biological replicates for each sample, respectively. A total of 40.81 Gb of clean data was obtained, the average GC content was 61.04%. The mapping results are shown in Supplementary Table S5. Gene expression levels between replicates for each sample exhibited high Pearson correlation coefficient values (more than 0.97), indicating good reproducibility between replicates.

Currently, FPKM is the most used commonly method to estimate gene expression levels. Based on the gene FPKM values, the expression level of genes with FPKM>60 in the reference transcriptome indicates high expression, while genes with FPKM of 0 ~ 1 were expressed at low levels or not expressed. The results showed that there were some differences in gene expression levels of pre-optimized BBD and CK 8947 genes (FPKM≥1) were expressed in BBD, accounting for 93.48% of the total number of genes, among which 3,383 genes were highly expressed (FPKM>60), accounting for 35.35% of the total number of genes; while 8,892 genes (FPKM≥1) were expressed in CK, accounting for 92.91% of the total number of genes. Among them, 3,173 genes were highly expressed (FPKM>60), accounting for 33.15% of the total number of genes. Further analysis of the control group CK and the experimental group BBD showed that 26 genes in the CK group were extremely highly expressed (FPKM>3,000) and 40 genes in the BBD group were extremely highly expressed (FPKM>2,000).

### KEGG pathway analysis

Sequences from 6 libraries were searched and annotated with the KEGG, GO, Swiss-Prot, Pfam, KOG, and NR public databases by Blast software. The percentages of annotated sequences based on the KEGG, GO, Swiss-Prot, Pfam, KOG and NR databases were 60.42, 69.72, 56.58, 70.32, 60.01, and 92.44%, respectively. KEEG classification results showed that 5,783 unigenes were enriched in 133 pathways. The main typical pathways are amino acid metabolism (360 genes), carbohydrate metabolism (548 genes) and lipid metabolism genes (120 genes). The amino acid metabolism subclass involves 13 pathways, namely (ko00220) arginine biosynthesis, (ko00250) alanine, aspartate and glutamate metabolism, (ko00260) glycine, serine and threonine metabolism, (ko00270) cysteine and methionine metabolism, (ko00280) valine, leucine and isoleucine degradation, (ko00290) valine, leucine and isoleucine biosynthesis, (ko00300) lysine biosynthesis, (ko00310) lysine degradation, (ko00340) histidine metabolism, (ko00350) tyrosine metabolism, (ko00360) phenylalanine metabolism, (ko00380) tryptophan metabolism, (ko00400) phenylalanine, tyrosine and tryptophan biosynthesis. The Carbohydrate metabolism subclass involves 15 pathways, especially including (ko00010) glycolysis/gluconeogenesis, (ko00020) citrate cycle (TCA cycle), (ko00500) starch and sucrose metabolism etc.

### Identification of differentially expressed genes

**Figure 2:**
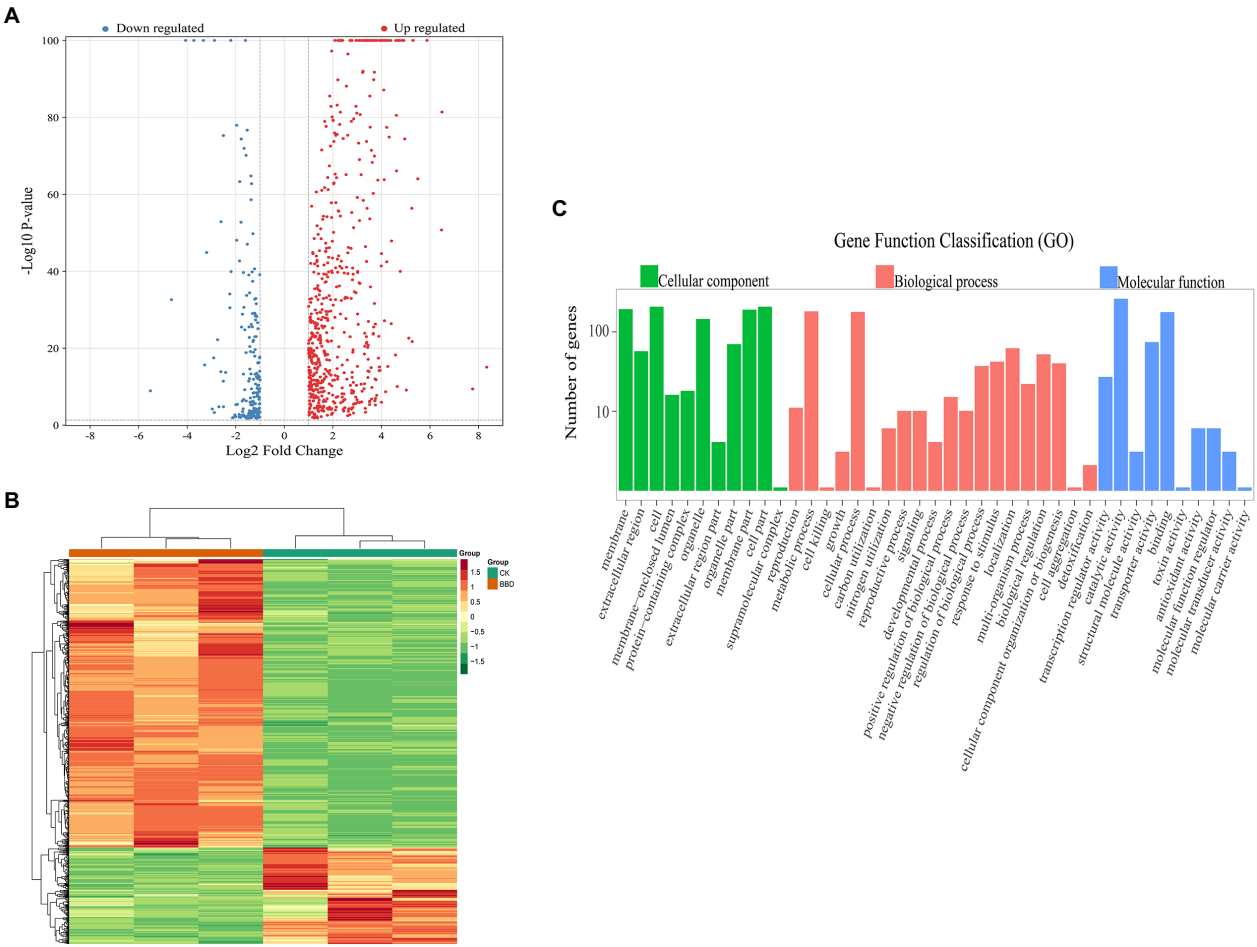
**(A)** Volcano plot of differentially expressed genes (DEGs); **(B)** Heatmap analysis of six samples; **(C)** Histogram of GO classification of DEGs. The abscissa represents the secondary GO entry and the ordinate represent the data of the differential genes of the GO entry.

Based on the threshold of |log_2_FC| ≥ 1 and FDR < 0.05, 790 significantly differentially expressed genes of *O. sinensis* mycelium were obtained when comparing BBD and CK, among which 592 genes were up-regulated and 198 genes were down-regulated. Among the differential genes within the threshold of|log_2_FC| ≥ 5, 12 genes with higher difference folds were up-regulated and one gene was down-regulated. Volcano plots that demonstrate significant DEGs between each comparison are shown in Figure 2A. The hierarchical clustering analysis for DEGs showed that the expression patterns of the two treatments were different (Figure 2B).

### Functional enrichment analysis of DEGs

#### Go functional classification

Based on the GO database, the functional classification of differentially expressed *O. sinensis* mycelium genes in BBD and CK sets can provide a macroscopic understanding of the functional distribution of differentially expressed genes (Figure 2C). The results show that a total of 790 DEGs were annotated to GO functional classifications, including biological processes, cellular component, and molecular functions. Among biological processes, there were 20 subtypes annotated, with most genes involved in metabolic processes (GO:0008152), cellular processes (GO:0009987), and localization (GO:0051179), with 181 (22.91%), 178 (22.53%), and 62 (7.84%), respectively. There are 11 subtypes in the category of cellular components, most of the genes were assigned to genes involved in cell (GO:0005623), a cellular fraction (GO:0044464), and membrane (GO:0016020), 207 (26.20%), 207 (26.20%) and 193 (24.43%), respectively. 10 subtypes were annotated in terms of molecular function, with most genes involved in catalytic activity (GO:0003824), binding (GO:0005488), and transporter protein activity (GO:0005215), with percentages of 261 (33.04%), 177 (22.41%), and 74 (9.37%), respectively.

#### KOG functional classification

**Figure 3:**
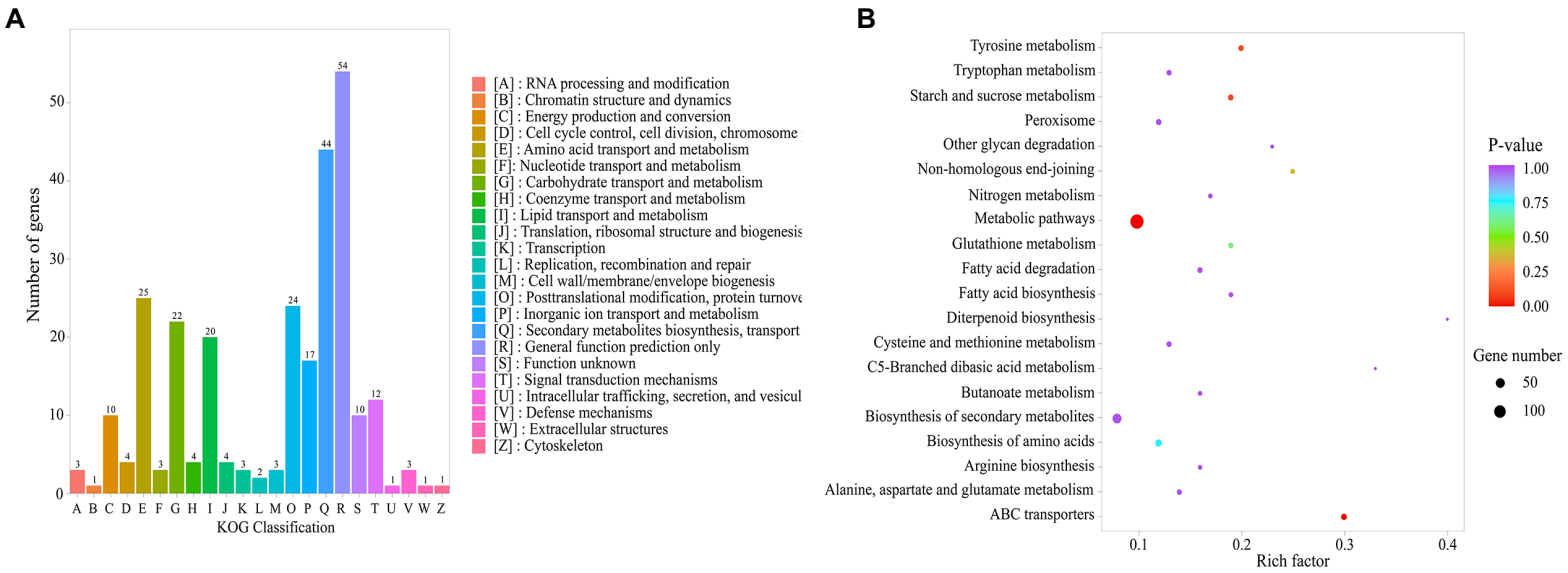
**(A)** A cluster of orthologous groups annotations of assembled DEGs. **(B)** KEGG enrichment of DEGs. Rich ratio = term candidate genes num/term genes num. *P*-value ≤0.05 indicates significant enrichment.

DEG functions were annotated based on the KOG (Clusters of orthologous groups for eukaryotic complete genomes) database. In this study, 271 (34.3%) DEGs were classified into 26 functional categories (Figure 3A). The main categories are general cluster general function prediction only (54, 19.93%), secondary metabolite biosynthesis, transport, and catabolism (44, 16.24%), amino acid transport and metabolism (25, 9.23%), posttranslational modification, protein turnover, chaperones (24, 8.86%), followed by carbohydrate transport and metabolism (22, 8.12%), lipid transport and metabolism (20, 7.38%), inorganic ion transport and metabolism (17, 6.27%), and signal transduction mechanisms (12, 4.43%).

#### KEGG functional classification

Based on the KEGG database, the DEGs were mapped into the KEGG database to perform enrichment analysis of metabolic pathways and further study their functions. The results showed that 200 DEGs were successfully annotated in 98 metabolic pathways. This study selected the top 6 metabolic pathways with significantly enriched differential genes (Figure 3B), including metabolic pathways (140, 70%), ABC transporters (13, 6.5%), starch and sucrose metabolism (11, 5.5%), tyrosine metabolism (10, 5%), non-homologous end-joining (6, 3%) and glutathione metabolism (8, 4%). To get a more comprehensive understanding of the expression status of differential genes before and after optimization of the medium, genes associated with increased mycelial protein content were screened based on transcriptomic data, the study concluded that the up-regulated genes were mainly present in metabolic pathways, ABC transporters, starch and sucrose metabolism, and tyrosine metabolism. However, it is noteworthy that 13 genes were enriched on ABC transporter proteins, which may be related to the increase in nutrient uptake by mycelium.

### Expression levels of DEGs involved in increased mycelial protein content

#### Amino acid metabolism pathway-related genes

**Figure 4:**
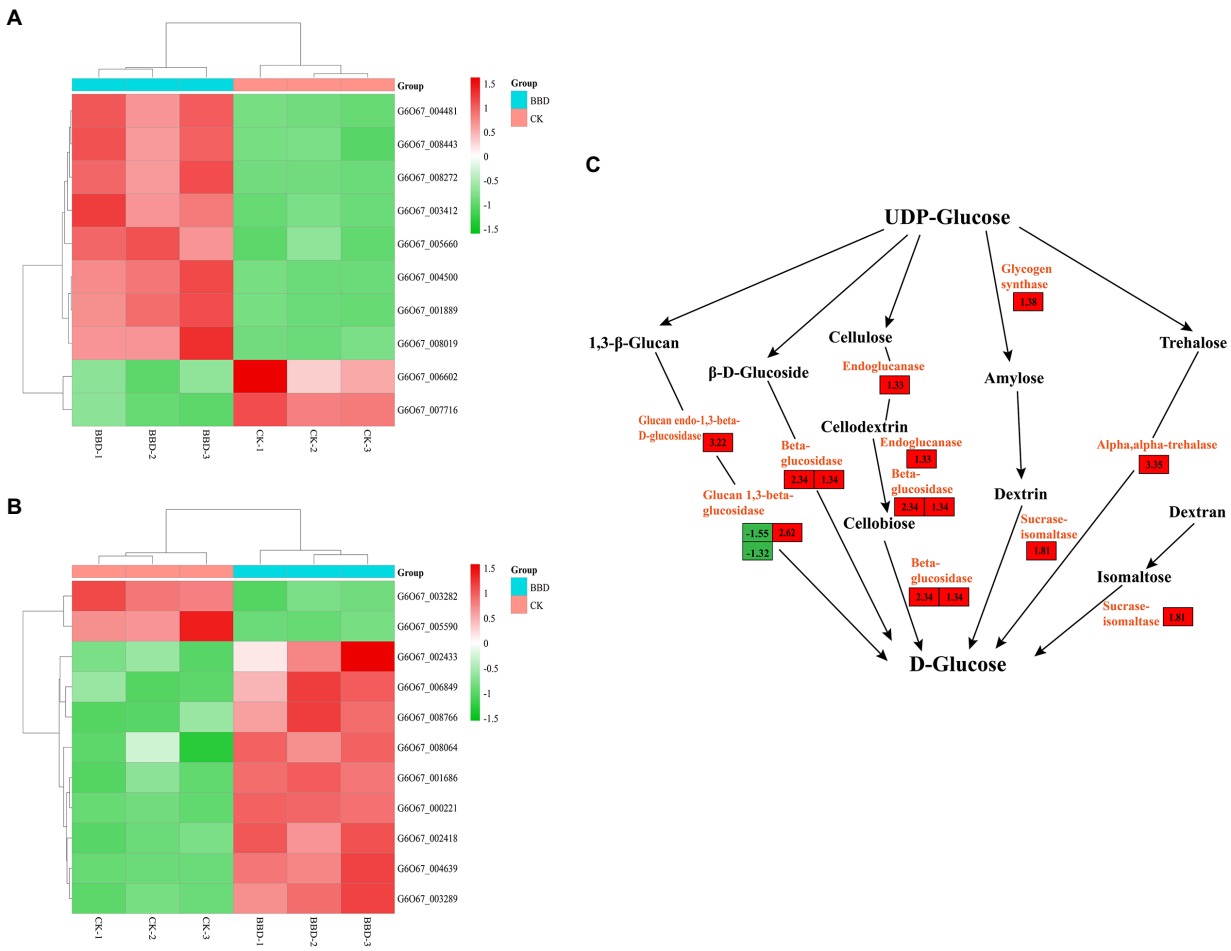
Based on KEGG analysis of differential genes in *O. sinensis* mycelium BBD and CK. Heatmaps illustrate the relative expression levels of these genes. **(A)** Tyrosine metabolism; **(B)** Starch and sucrose metabolism; **(C)** DEGs in starch and sucrose metabolism.

Based on the transcriptome data of *O. sinensis*, it was found that most DEGs were enriched in amino acid metabolic pathways, such as tyrosine metabolism, glutathione metabolism, cysteine and methionine metabolism, alanine, aspartate and glutamate metabolism. This means that the optimization of medium stimulates mycelium growth and development and substance synthesis. The tyrosine metabolic pathway was significantly enriched in the amino acid metabolic pathway (Figure 4A). Notably, tyrosinase and catechol-*O*-methyltransferase played important roles as significantly upregulated enzymes in the tyrosine metabolic pathway. Some DEGs annotated as tyrosinase (G6O67_008272/G6O67_003412/G6O67_005660/G6O67_008019) were considerably more expressed in BBD than in CK. Previous study has indicated that tyrosinase catalyzes the oxidation of tyrosine to two catecholamines (dopamine and dopamine), which are key enzymes in melanin synthesis and control the activity of melanocytes in living organisms (Sánchez Ferrer et al., 1995; Masuoka and Maekawa, 2016). Besides the effect from mycelial protein content, the difference in mycelial pigmentation of BBD and CK might be indirectly related to tyrosinase (Seo et al., 2003). Furthermore, some DEGs were annotated as homogentisate 1,2-dioxygenase (*HGD*), alcohol dehydrogenase 1/7 alcohol dehydrogenase 1/7 (*ADH1_7*) were both upregulated. It is noteworthy that the primary-amine oxidase (*AOC3*) was significantly down-regulated.

DEGs related to glutathione metabolism γ-glutamylcyclo-transferase (G6O67_001865), γ-glutamyl transpeptidase/glutathione hydrolase (G6O67_004614), isocitrate dehydrogenase (G6O67_006510) and glutathione S-transferase (G6O67_008888, G6O67_000596, G6O67_001875, G6O67_005094) were significantly up-regulated. Their final products are 5-oxoproline, L-cysteine, glutathione etc. Research has shown that fungi are high in bioactive peptides or amino acids such as glutathione and L-cysteine, which are useful for immunity, antioxidant, detoxification, and cancer treatment (Lv et al., 2019; Minich and Brown, 2019). Additionally, L-cysteine is an essential sulfur-containing amino acid for the synthesis of reduced glutathione. Glutathione S-transferase (G6O67_008888, G6O67_000596, G6O67_001875, G6O67_005094) was found to be the most significantly upregulated gene expressed in BBD. Previous studies have reported that GSTs are a major detoxifying enzyme in organisms, catalyzing the binding of glutathione to electrophilic substrates, increasing the water solubility of toxic substances, and eliminating reactive oxygen species produced in organisms subjected to oxidative stress (Morel et al., 2009). Therefore, glutathione s-transferase may be an important factor leading to the increase of mycelial active protein content, improving the antioxidant function of mycelium.

In alanine, aspartate and glutamate metabolism, most of enzymes containing adenylosuccinate synthase (*purA*), glutamate dehydrogenase (*GDH*), glutamine synthetase (*glnA*), glutamine-fructose-6-phosphate transaminase (*glmS*) and carbamoyl-phosphate synthase (*CAD*) were significantly upregulated. It was found that the upregulation of glutamate dehydrogenase and glutamine synthetase accelerates the synthesis of glutamate and glutamine during the synthesis of amino acids. As a major metabolic center in many organisms, glutamate, in addition to its role in protein synthesis, is involved in a variety of processes such as amino acid synthesis and the formation of secondary natural products and nitrogen assimilation (Walker and van der Donk, 2016). Glutamate is usually produced through two pathways, both of which lead to the overall conversion of 2-oxoglutarate (an intermediate of the citric acid cycle) to glutamate. One of these pathways is the reduction of ammonia as a nitrogen donor through glutamate dehydrogenase (*GDH*) to aminate 2-oxoglutarate (Brosnan and Brosnan, 2013). *GDH* belongs to the family of amino acid dehydrogenases, with other members acting on leucine, phenylalanine or valine, and tends physiologically in the direction of deamination (Tang and Hutchinson, 1993). Additionally, glutamine is produced by the action of glutamine synthetase, which uses ammonium as a nitrogen source to catalyze the ATP-dependent amination of glutamate. This finding implies that, because of the upregulation of glutamate dehydrogenase in relation to glutamate and glutamine synthesis, the variety of amino acids synthesized after medium optimization may be more in the non-optimized medium.

#### Synthetic D-glucose-related genes

To dig deeper into the relevant genes affecting mycelial protein content, the DEGs annotated in KEGG based on the starch and sucrose metabolic pathway were first targeted and a total of 11 differential genes were found, nine of which were up-regulated and two down-regulated (Figure 4B). Six pathways for the synthesis of D-glucose were found to exist in this pathway (Figure 4C). The largest number of genes annotated was β-glucosidase, which is an important component of the cellulolytic enzyme family. It mainly catalyzes the generation of cellobiose from cellulose dextrin and then is catalyzed by β-glucosidase to glucose. Meanwhile, β-glucosidase can also catalyze the generation of glucose from β-D-glucoside. During optimization, β-glucosidase was higher than the unoptimized level. Other differential genes were annotated as endoglucanases, which often work together with β-glucosidase to completely hydrolyze cellulose molecules into glucose. Therefore, we speculate that the differential genes may provide an additional carbon source for the improved protein content of the optimized mycelium.

#### Other mycelial protein content-related genes

After transcriptome data analysis, this study found that DEGs were mainly enriched in four major categories involving metabolism, genetic information processing, environmental information processing and cellular processes. Among the subclass of the carbohydrate metabolism pathway, this research particularly focused on the Citrate cycle (TCA cycle) (Eprintsev et al., 2018). Notably, pyruvate carboxylase (*pyc*), isocitrate dehydrogenase (*IDH*) and citrate synthase (*gltA*) were significantly upregulated in the TCA cycle. Citrate synthase and isocitrate dehydrogenase are the rate-limiting enzymes in the TCA cycle (Akram, 2014). They are essential for the intermediate products of the cycle (e.g., oxaloacetate, α-ketoglutarate), which are the raw materials for the synthesis of sugars, amino acids and fats (Fernie et al., 2004; Xie et al., 2022). Isocitrate dehydrogenase has important oxidoreductase and decarboxylation functions as a component of the TCA cycle. It catalyzes the decarboxylation of isocitrate to produce α-ketoglutarate, releasing CO_2_ and NADPH (Spaans et al., 2015). Previous studies have also demonstrated that isocitrate dehydrogenase is a major source of NADPH and has an active role in the synthesis of metabolites such as amino acids (Becker et al., 2007; Papagianni, 2012). In addition, *IDH* plays an important role in cellular defense against oxidative damage and ROS detoxification (Mailloux et al., 2007; Paul et al., 2007). The presence of these enzymes suggests that the gradual increase in protein content after medium optimization probably influences the promotion of the tricarboxylic acid cycle in mycelium.

#### RT-qPCR validation analysis of DEGs

**Figure 5:**
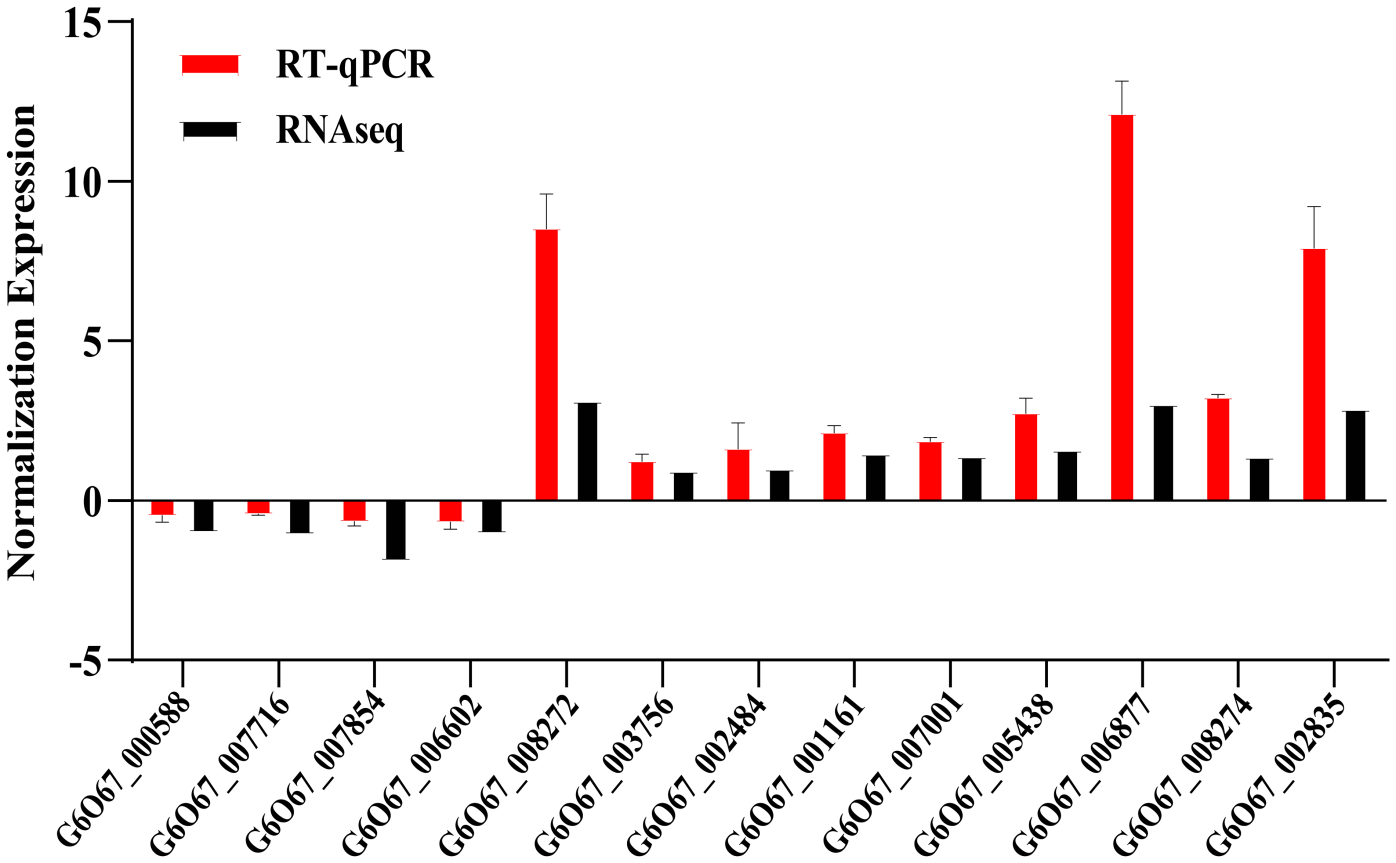
Quantitative identification of differentially expressed genes. Red bars represent RT-qPCR results (2^−ΔΔCt^). Black bars indicate RNA-seq results (log2 fold change). Error bars represent standard errors. *ACT1* gene was the internal reference.

To confirm the reliability of the RNA-Seq analysis, RT-qPCR analysis of the expression levels of randomly selected genes in the major pathways enriched by DEGs was performed in this study. RT-qPCR was performed on 13 genes to verify differential gene expression obtained by RNA-Seq (Figure 5). This result found that the direction of fold change detected by RT-qPCR of gene expression changes was similar to Illumina sequencing results, with four genes down-regulated and nine genes up-regulated. These outcomes demonstrate the dependability of our transcriptome data.

## Discussion

### Effect of different medium components on mycelial protein content of *Ophiocordyceps sinensis* under response surface methodology

Fungal products from *O. sinensis* are an important alternative to Chinese Cordyceps. The main ingredient found to have a good development prospect is Cordyceps protein, and its level can also be used as an important index for quality evaluation (Wang et al., 2018). Therefore, it is crucial to gain a reliable culture optimization method to improve the enzymatic activity and total protein of mycelium. Traditional methods for optimization of culture media often use an orthogonal test method and response surface method, which can be used to consider several factors simultaneously and find the best combination of factor levels but cannot obtain a clear regression equation of factors and response values over the whole region (Kim and Na, 1997; Zhang et al., 2010). However, PBD and RSM can be used to determine the influence of the test factors and the interaction between the factors on the response values during the treatment (Elibol, 2002). A previous study using PBD and CCD design has shown the optimal concentrations of NaH_2_PO_4_, Na_2_HPO_4_, NaCl, yeast extract and corn pulp, which resulted in the highest productivity of succinic acid (Stylianou et al., 2021). In addition, it has been found in previous studies that the use of PBD and RSM for media optimization are very effective methods and produce satisfactory results (Guo et al., 2018).

In this research, the medium of *O. sinensis* was optimized using RSM, which included medium components containing a carbon source, nitrogen source and trace elements. It was found that a carbon source contributed the most to the increase of mycelial protein content of *O. sinensis*. Carbohydrates have a great influence on the fermentation process when the microorganisms undergo mass multiplication by fermentation culture. Sufficient carbon must be available for biosynthesis and energy production to allow for the survival of the cells (Manirafasha and Lu, 2015). It was found that in the culture of *O. sinensis*, carbon and nitrogen sources are the main building block sources for protein synthesis, and appropriate changes in the ratio have an effect on mycelium production and active proteins or enzymes (Dong et al., 2009). In the three-dimensional surface plot and contour plot of the interaction effects, indicating two factors that are close to elliptical and have significant interaction are beef broth and glucose. These provide a carbon source, and their ratios play an important role in the growth of the fungi. When the addition of peptone was 0.1%, the mycelial protein content increased and then decreased with the addition of both, with the same trend, but the contour density along the direction of beef broth was greater than that along the direction of glucose, indicating that increasing the amount of beef broth was more effective than increasing glucose within a certain range.

It is noteworthy that the addition of glucose at the highest mycelial protein content during medium optimization gives an intermediate value, indicating that too much or too little may hinder the growth of mycelium. It was found that the combination of medium components had a profound effect on the metabolic pathways of microorganisms regulating the production of many metabolites. This implies that the metabolic pathways of mycelia cultured under the optimized medium are active when the glucose concentration reaches 2%. This is consistent with the results of the transcriptome analysis.

### Transcriptome analysis before and after mycelium media optimization

The statistical results support the view of this study in the RSM hypothesis that carbohydrates play an important role in the transcriptome. Transcriptome analysis revealed that DEGs are associated with GO terms ‘metabolic processes’, ‘cellular processes’ and ‘catalytic activity’. This implies that the expression of metabolic processes in altered medium composition is associated with mycelial dynamics and that proteins or enzymes expressed by DEGs contribute to medium utilization by the mycelium (Xie et al., 2022). Increased mycelial protein content implies an increase in protein or protein mixtures produced by the fungus and excreted to the culture medium. Increased mycelial protein content usually manifests as, for example, an increased level of enzyme activity, such as cellulase, hemicellulase, protease, or production of more total extracellular protein (Pakula et al., 2013). Previous studies have shown that cellulase, hemicellulase, ligninase, and pectinase production is susceptible to regulation primarily at the transcriptional level in filamentous fungi (Aro et al., 2005).

KEGG enrichment of DEGs showed that they are mainly involved in ATP binding cassette (ABC) transport proteins, ‘starch and sucrose metabolism’, ‘amino acid metabolism’ (e.g., alanine, aspartate and glutamate metabolism, tyrosine metabolism), fatty acid degradation, and fatty acid biosynthesis. These pathways are associated with basic metabolic types and are involved in the synthesis of primary and secondary metabolites. Previous studies have shown that starch and sucrose metabolism has a key role in fungal growth (Chen et al., 2021). Carbohydrates are important components of fungal cell walls and fungal growth makes higher metabolic demands (Coronado et al., 2008). Nine genes involved in the “starch metabolism process” were significantly up-regulated and two were significantly down-regulated. Annotation showed DEGs encoding a glucan endo−1,3-beta-D-glucosidase (G6O67_004639), an endoglucanase (G6O67_001686), and DEGs encoding alpha-trehalase (G6O67_008766) in this transcriptome. Endoglucanase is one of the components of cellulase, which hydrolyzes the β − 1,4-glycosidic bond in the internal non-crystalline region of the cellulose molecule, ultimately hydrolyzing cellulose to glucose, providing the fungus with more carbon and access to more exogenous proteins (Pakula et al., 2013). Not only did the sucrose and starch pathways contribute to the increase in mycelial protein content during medium optimization, but starch and sucrose metabolism are associated with the production of energy and active compounds for fruiting body maturation (Tong et al., 2020).

Notably, the 11 upregulated genes were enriched in five ATP-binding cassette (ABC) transporter protein subfamilies, namely ABCA (ABCA3), ABCB (ABCB1), ABCC (ABCC1), ABCD (PXA1/2), ABCG (PDR5, SNQ2), which were found in previous studies to be possibly related to nutrient uptake by the fungus (Adour et al., 2010; Wu et al., 2022). GO analysis showed that the transporter proteins are involved in carbohydrate derivative binding, ATP binding and have active transmembrane transporter protein activity. The increased expression of ATP-binding cassette (ABC) transport proteins could facilitate the transport of glucose, which requires entry into the cytoplasmic membrane (Frelet and Klein, 2006). In the mycelium culture, the optimized medium composition may have activated the ABC transporter proteins to using the energy generated by ATP hydrolysis to transport the substrate through a reverse concentration gradient. In addition, ABC transport proteins are also involved in cellular processes such as DNA repair, transcription and regulation of gene expression (Braz et al., 2004). Three transport proteins are particularly highly expressed in sclerotia and show the positive effects on transport proteins and the diffusion of specific molecules such as peptides and proteins (Zhao et al., 2020). The highly expressed transporter proteins provide the conditions for substrate transport and contribute to the growth and development of mycelium and protoplasts.

Optimization of culture substrates or genetic modification of fungal strains are commonly used as the main ways of improving the protein production of filamentous fungi (Punt et al., 2002). Overexpression or deletion of genes is used to improve protein and enzyme yields. Yoon et al. disrupted decuple protease gene in the filamentous fungus *Aspergillus oryzae* to greatly improve heterologous protein production (Yoon et al., 2010). In addition, Genetic modifications can also be used to improve heterologous protein production when promoters or regulatory elements of genes encoding secreted proteins are used for heterologous expression (Pakula et al., 2013). Madhavan et al. demonstrated that after disrupting their natural β-galactosidase (*lac4*) promoter the fungal promoter was shown to have the potential to drive heterologous protein production in *Kluyveromyces lactis* (Madhavan and Sukumaran, 2014). The expression level of recombinant Pro-transglutaminase (pro-MTG) from *Streptomyces mobaraensis* achieved 87.6 U/ml and 70.7 U/ml under the control of two constitutive promoter P*_sodA_* and P*_ydzA_*, respectively, demonstrating that optimized strong promoters can express extracellular proteins at high levels in *Bacillus subtilis* (Liu et al., 2018). However, this study identified DEGs and major enrichment pathways, but the use of promoters and modification of the genes has not been explored. Further attention and efforts are needed to explore the effects of genetic modifications of the fungus and the conditions of protein production in *O. sinensis* modified under suitable media.

## Conclusion

In summary, this work used Plackett-Burman design, path of steepest ascent method, and Box–Behnken design to screen the culture medium of *O. sinensis* and obtained the best medium with the highest mycelial protein content, while transcriptomics was used to analyze the medium before and after optimization at the gene level. It found that changing the ratio of the medium components could increase the mycelial protein content of *O. sinensis*. However, excessive addition would inhibit protein synthesis. The micronutrients KH_2_PO_4_ and MgSO_4_ in the medium had no significant effect on the total mycelium protein content (*p* > 0.05). The highest mycelial protein content of 2.11% was obtained based on the optimization results, at which point the optimized medium formulation was finalized as 20% beef broth, 0.10% peptone, 2% glucose, 0.15% yeast extract, 0.20% KH_2_PO_4_, and 0.02% MgSO_4_. Most of the up-regulated differential genes affecting mycelium protein content were analyzed from a transcriptomic perspective. The identification of DEGs revealed 790 genes, of which the number of up-regulated genes was 592 and the number of down-regulated genes was 198 genes. The optimized expression pattern had more up-regulated genes than before optimization. The main enriched pathways of DEGs affecting protein content were ABC transporters, starch and sucrose metabolism, tyrosine metabolism and glutathione metabolism. In addition, it is noteworthy that individual enzymes such as tyrosinase (*TYR*), glutathione S-transferase (*gst*), glutamine synthetase (*glnA*), β-glucosidase, pyruvate carboxylase (*pyc*), isocitrate dehydrogenase (*IDH1*) and citrate synthase (*gltA*) potentially contribute more to the increase in mycelial protein content, while the difference in mycelium color before and after medium optimization was likely due to tyrosinase. In conclusion, this study not only lays a foundation for further research targeting the proteins of *O. sinensis*, but also benefits industrialization and development applications of *O. sinensis*.

## Data availability statement

The datasets presented in this study can be found in online repositories. The names of the repository/repositories and accession number(s) can be found in the article/Supplementary material.

## Author contributions

C-YT designed and performed the experiments and wrote the manuscript. X-ZL, WS, XL, and JW analyzed the interpretation of the data-designed experiments and revised them critically for intellectual content. J-BC, JL, and TW performed the experiments. Y-LL provided reagents and experimental equipment. All authors contributed to the article and approved the submitted version.

## Funding

This research was funded by a grant of the major science and technology projects of Qinghai Province (2021-SF-A4).

## Conflict of interest

The authors declare that the research was conducted in the absence of any commercial or financial relationships that could be construed as a potential conflict of interest.

## Publisher’s note

All claims expressed in this article are solely those of the authors and do not necessarily represent those of their affiliated organizations, or those of the publisher, the editors and the reviewers. Any product that may be evaluated in this article, or claim that may be made by its manufacturer, is not guaranteed or endorsed by the publisher.

## Footnotes

^1^ https://www.ncbi.nlm.nih.gov/genome/11415?genome_assembly_id=892900

^2^ https://www.genome.jp/kegg
