# Supplementary material for: Medium optimization for high mycelial soluble protein content of Ophiocordyceps sinensis using response surface methodology
Source: Front Microbiol. 2022 Dec 9;13:1055055. doi: 10.3389/fmicb.2022.1055055 (PMC9780674; doi:10.3389/fmicb.2022.1055055)
Supplement: Supplementary file 1 [file Table_1.docx]

**Supplementary Table 1.** Range of different factors investigated by the PB design.

| Symbols | Variables | Lower/% (-1) | Higher/% (1) |
| --- | --- | --- | --- |
| X_1_ | beef broth | 30 | 50 |
| X_2_ | peptone | 0.30 | 0.70 |
| X_3_ | yeast extract | 0.10 | 0.25 |
| X_4_ | glucose | 1 | 4 |
| X_5_ | KH₂PO_4_ | 0.10 | 0.40 |
| X_6_ | MgSO_4_ | 0.01 | 0.04 |

**Supplementary Table 2.** Design and results of steepest ascent method.

| Treatment | beef broth (%) | peptone (%) | glucose (%) |
| --- | --- | --- | --- |
| 1 | 20 | 0.1 | 1 |
| 2 | 30 | 0.3 | 2 |
| 3 | 40 | 0.5 | 3 |
| 4 | 50 | 0.7 | 4 |
| 5 | 60 | 0.9 | 5 |

**Supplementary Table 3.** Box-Behnken design with experimental.

| Factor | Variables | Levels (%) | | |
| --- | --- | --- | --- | --- |
|  |  | -1 | 0 | 1 |
| X_1_ | Beef broth | 20 | 30 | 40 |
| X_2_ | Peptone | 0.1 | 0.3 | 0.5 |
| X_3_ | Glucose | 1 | 2 | 3 |

| Run | Coded levels of variables | | |
| --- | --- | --- | --- |
|  | X1 | X2 | X3 |
| 1 | 0 | 1 | -1 |
| 2 | 0 | 0 | 0 |
| 3 | -1 | 0 | -1 |
| 4 | 0 | -1 | -1 |
| 5 | -1 | 0 | 1 |
| 6 | 0 | 0 | 0 |
| 7 | -1 | 1 | 0 |
| 8 | 1 | 0 | -1 |
| 9 | 1 | 0 | 1 |
| 10 | 0 | 0 | 0 |
| 11 | 1 | 1 | 0 |
| 12 | 0 | 1 | 1 |
| 13 | 0 | -1 | 1 |
| 14 | 0 | 0 | 0 |
| 15 | 0 | 0 | 0 |
| 16 | 1 | -1 | 0 |
| 17 | -1 | -1 | 0 |

**Supplementary Table 4.** RT-qPCR primers for DEGs in *O. sinensis* and the internal control gene

| Gene ID | Primer | sequence（5’-3’） |
| --- | --- | --- |
| ACT1 | Forward primer | CAATCGGCACAACTGGACA |
|  | Reverse primer | GACGACCTGAGCGGAATA |
| G6O67_000588 | Forward primer | GCACCAAATCGAGCAAGAAG |
|  | Reverse primer | CCTTGGTCGAGGTCAATGT |
| G6O67_007716 | Forward primer | CGGAAGAACACCCGAACTTAT |
|  | Reverse primer | CAGTCTCTCCACCATCTTTCAG |
| G6O67_007854 | Forward primer | GCCAGAGGAAAGGGCAAATA |
|  | Reverse primer | TTTCCCTTCGGCGTTGTATC |
| G6O67_006602 | Forward primer | CAACTGGAAGCACAACAACTG |
|  | Reverse primer | GTCGAACTTGAAGGCGAAGA |
| G6O67_008272 | Forward primer | GGATGGGACCTGTTTCTTCTT |
|  | Reverse primer | GTGGATACCGGCGATTTGATA |
| G6O67_003756 | Forward primer | CATTCTCAAGTTTCTCGGCTTTG |
|  | Reverse primer | GTATCTCGGCGTCACTCTTG |
| G6O67_002484 | Forward primer | CGTCGCATCATCTTCATTGC |
|  | Reverse primer | CTCGACCGAGATTGGGATTT |
| G6O67_001161 | Forward primer | CGGCCATCATCATCTCCTTT |
|  | Reverse primer | CCTCTTGATCCACACCTTGAT |
| G6O67_007001 | Forward primer | TCCACACCGTCGTCATTTC |
|  | Reverse primer | GCAGGTATGGTCTGCTTGAT |
| G6O67_005438 | Forward primer | ATGATGGCTGCCTGTCAAA |
|  | Reverse primer | CGATGAAGATGTGGAGAGTGTAG |
| G6O67_006877 | Forward primer | CTACCAGTACGTTGACGAGTATTT |
|  | Reverse primer | CGCTCCCACTTATCATCGTATC |
| G6O67_008274 | Forward primer | CTGAAGCTCAAGGAGTGGTATG |
|  | Reverse primer | CAAAGTAGGCGAGATGGAGATAG |
| G6O67_002835 | Forward primer | GTGTTTGGCGACGACTTTG |
|  | Reverse primer | ACAGTCTCGATGTAGCGTTTC |

**Supplementary Table 5.** Sequencing data quality assessment

| Sample | Raw Reads | Clean Reads | Clean Base (G) | Error Rate (%) | Q20 (%) | Q30 (%) | GC Content (%) | Reads mapped | Mapped ratio |
| --- | --- | --- | --- | --- | --- | --- | --- | --- | --- |
| CK1 | 45241528 | 42395048 | 6.36 | 0.03 | 97.84 | 94.47 | 60.90 | 40509161 | 95.55% |
| CK2 | 51459806 | 48196122 | 7.23 | 0.02 | 97.91 | 94.67 | 60.91 | 46040040 | 95.53% |
| CK3 | 48522936 | 46231214 | 6.93 | 0.02 | 97.92 | 94.65 | 60.91 | 44207540 | 95.62% |
| BBD1 | 48019372 | 45300060 | 6.80 | 0.03 | 97.81 | 94.41 | 61.11 | 43503264 | 96.03% |
| BBD2 | 46260320 | 43604104 | 6.54 | 0.02 | 97.9 | 94.63 | 61.17 | 41908709 | 96.11% |
| BBD3 | 49425530 | 46331164 | 6.95 | 0.03 | 97.81 | 94.41 | 61.21 | 44449986 | 95.94% |
